# Phage quest: a beginner’s guide to explore viral diversity in the prokaryotic world

**DOI:** 10.1093/bib/bbaf449

**Published:** 2025-09-03

**Authors:** Carolin Charlotte Wendling, Marie Vasse, Sébastien Wielgoss

**Affiliations:** Department of Environmental Systems Science, Institute of Integrative Biology, ETH Zürich, Universitätstrasse 16, 8092 Zürich, Switzerland; Max von Pettenkofer-Institut, Ludwig-Maximilians-Universität (LMU), Pettenkoferstraße 9a, 80366 München, Germany; Department of Environmental Systems Science, Institute of Integrative Biology, ETH Zürich, Universitätstrasse 16, 8092 Zürich, Switzerland; CNRS UMR 5164, ImmunoConcept, Université de Bordeaux, Site de Carreire, Bâtiment BBS, 2 Rue Dr Hoffmann Martinot, 33076 Bordeaux Cedex, France; Department of Environmental Systems Science, Institute of Integrative Biology, ETH Zürich, Universitätstrasse 16, 8092 Zürich, Switzerland

**Keywords:** bacteriophages, prophages, metagenomics, microbial bioinformatics, gene annotation, phage prediction

## Abstract

The increasing interest in finding new viruses within (meta)genomic datasets has fueled the development of computational tools for virus detection and characterization from environmental samples. One key driver is phage therapy, the treatment of drug-resistant bacteria with tailored bacteriophage cocktails. Yet, keeping up with the growing number of automated virus detection and analysis tools has become increasingly difficult. Both phage biologists with limited bioinformatics expertise and bioinformaticians with little background in virus biology will benefit from this guide. It focuses on navigating routine tasks and tools related to (pro)phage detection, gene annotation, taxonomic classification, and other downstream analyses. We give a brief historical overview of how detection methods evolved, starting with early sequence-composition assessments to today’s powerful machine-learning and deep learning techniques, including emerging language models capable of mining large, fragmented, and compositionally diverse metagenomic datasets. We also discuss tools specifically aimed at detecting filamentous phages (*Inoviridae*), a challenge for most phage predictors. Rather than providing an exhaustive list, we emphasize actively maintained and state-of-the-art tools that are accessible via web or command-line interfaces. This guide provides basic concepts and useful details about automated phage analysis for researchers in different biological and medical disciplines, helping them choose and apply appropriate tools for their quest to explore the genetic diversity and biology of the smallest and most abundant replicators on Earth.

## Introduction

**Figure 1:**
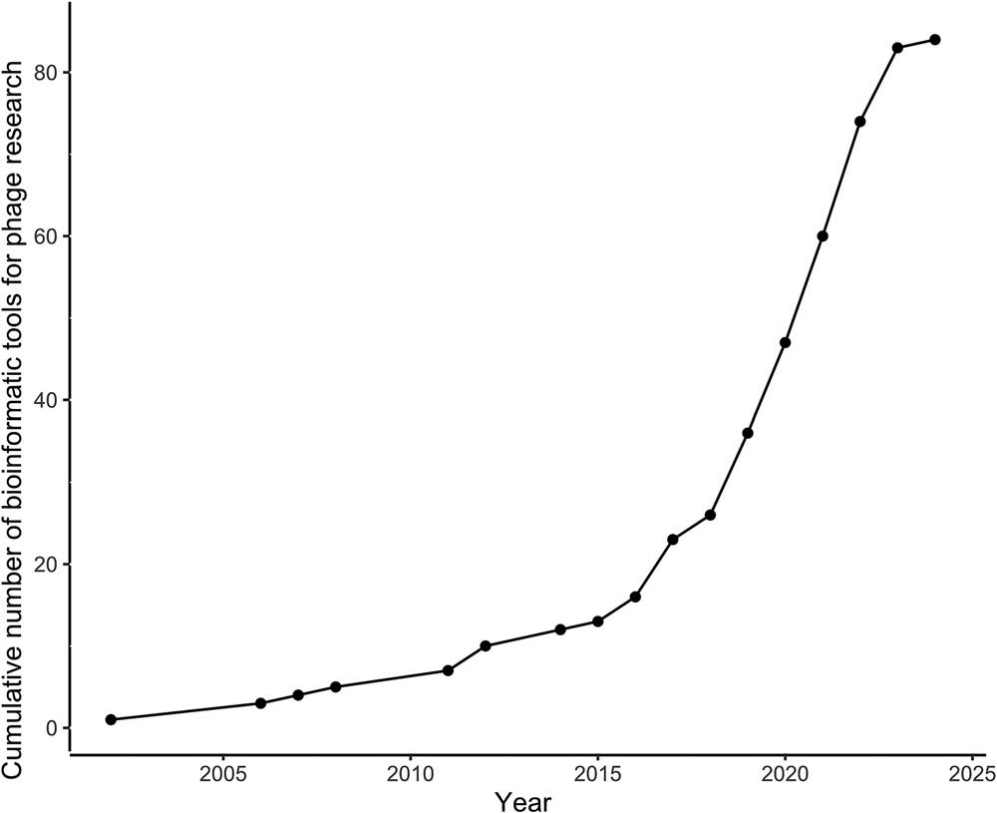
Surging interest in computational phage research. The chart depicts the cumulative count of all bioinformatic tools referenced in this review, covering methods of phage detection, annotation, taxonomic classification, host prediction, life cycle inference, and genome quality assessment.

The growing interest in discovering new viruses in (meta)genomic datasets has led to a rapid increase in newly developed computational tools for virus detection and characterization from environmental samples [1–6]. This interest is also sparked by the potential of phage therapy, the application of phages to treat bacterial infections, especially those involving drug-resistant bacteria [7]. However, this surge of interest in bacteriophages (phages) extends beyond the promise of medical applications and is based on recognizing our planet as a bacterial world [8], where phages play pivotal roles as the most abundant replicators [9], shaping ecological and evolutionary dynamics of microbial communities, and have cascading effects on plants, animals, and entire ecosystems [10]. As a result of ever-cheaper sequencing costs, researchers from diverse fields, including microbiology, medicine, ecology, and evolution, started to explore and identify phages either in their own (meta)genomic datasets or in publicly available databases. With the rapid pace at which new virus analysis tools are emerging in recent years (Fig. 1), it becomes increasingly more difficult for researchers to select the most appropriate approaches to answer their most relevant questions. Acknowledging these dynamics, here we provide a comprehensive guide that equips researchers with the necessary knowledge to detect and describe bacterial viruses in genomic and metagenomic datasets, enabling an easy entry into this rapidly evolving research field. While recent reviews were aimed at technical users in metagenomics [11], this guide is aimed at both wet-lab phage biologists with limited bioinformatics expertise and bioinformaticians with little background in virus biology.

We begin with key concepts in bacteriophage biology and then briefly introduce the computational principles behind phage detection. We then provide a historical account of the evolution of (pro)phage detection tools and highlight modern state-of-the-art algorithmic approaches. After that, we transition to the core part of our review, a step-by-step guide comprising four parts. In these steps, we cover popular and well-maintained tools without claiming to be exhaustive and include methods that support the detection of filamentous phages (*Inoviridae*), an oft-neglected group that includes the important representative phage M13. Data processing, genome assembly, phylogenetics, and comparative genomics are only briefly addressed, as they fall outside the scope of this review.

## Key concepts in bacteriophage biology

**Figure 2:**
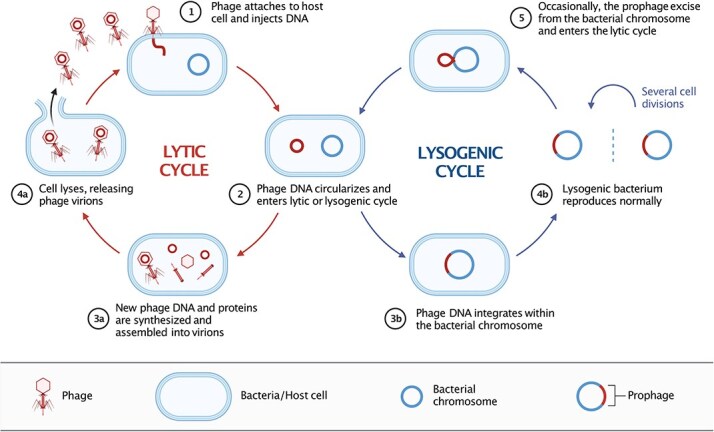
Illustration of lytic and lysogenic bacteriophage life cycles. In both cycles, the phage binds to the bacterial cell (1) and the phage’s genetic material then enters the host cell (2). During the lytic cycle, the phage multiplies (3a) and releases mature viruses through host cell lysis (4a). The lysogenic cycle is characterized by phage genome integration into the bacterial genome (prophage formation, 3b), vertical inheritance through host replication (4b), and occasional phage genome excision (5) before entering the lytic cycle (3a-4a). Created in BioRender. Wielgoss, S. (2025) https://BioRender.com/4si2h1x

Phages display remarkable diversity in genome structure, morphology, and life cycle strategies [12]. Their genomes are encoded as either single- or double-stranded DNA or RNA and are often enclosed in protein shells, either spherical capsids or filamentous coats. Beyond morphology, phages evolved different life cycles (Fig. 2): virulent phages kill their infected hosts via the lytic cycle, ensuring rapid horizontal transmission. Temperate and filamentous phages establish long-term associations with their bacterial hosts, ensuring vertical host transmission. While filamentous phages typically persist extrachromosomally without causing lysis, temperate phages insert their genetic material into the host genome and together form the lysogen. Insertion happens either at specific attachment sites via integrases (phage lambda) or at random via transposases (phage mu). Integrated prophages can exit the host genome spontaneously or in response to stress via a molecular switch, replicate, and subsequently lyse the host. Prophages are widely found in bacterial genomes and can constitute up to a fifth of the host genome [13]; e.g. *Escherichia coli* O157:H7 strain Sakai harbors 18 prophages [14]. Integrated phages sometimes lose their ability to switch to the lytic cycle or produce viable viral particles following the acquisition of deleterious mutations [15]. Finally, more complex, multipartite viruses exist that are distributed across different genomic segments, each encapsulated in separate particles [16]. One example is RNA-phage phi-6, which infects *Pseudomonas phaseolicola* [17]. Such a complicated genome organization represents an important challenge for phage prediction tools.

## Principles of automated phage prediction

Viral signals can be successfully detected with different computational approaches (Table 1). These approaches differ primarily in how much they rely on similarity to known viral sequences.

- *Sequence-similarity-based approaches* identify viral regions by homology to known phage proteins, e.g. via BLAST or hidden Markov models (HMMs) profiles from databases such as the prokaryotic virus orthologous groups (pVOGs) database. These tools often use sliding windows to detect phage-like regions enriched for phage genes. These methods are strongly dependent on database completeness and can miss divergent or unknown phages.
- *Hybrid approaches* integrate classical sequence-similarity-based methods with sequence-agnostic approaches. The latter is based on homology-independent features, i.e. GC/AT skew, transcription directionality, gene length, or tRNA occurrence, to achieve higher accuracy and flexibility. Many recent hybrid tools incorporate machine or deep learning (ML/DL) to enhance the detection of fragmented and novel genomes.
- *K-mer-based methods* classify sequences using the frequency of short nucleotide genomic substrings of length *k* (*k-mers*), equivalent to “DNA words”. This allows detection of viruses with limited similarity to known phages. These methods are alignment-free and can handle genome rearrangements but are sensitive to different *k-mer* sizes and input quality.
- *ML and DL approaches* apply data-driven models to detect unknown or less well-characterized viruses, including ssDNA viruses [18]. ML/DL-models learn complex patterns that distinguish viral from microbial and plasmid sequences. Common ML-models are random forests (RF), a range of learning methods that build multiple decision trees and combine their output for predictions, and support vector machines (SVMs), which identify the optimal boundaries between different groups in the data. Widely used DL-models are convolutional neural networks (CNNs), which excel at identifying local data patterns (including *k-mer* frequencies), and long short-term memory (LSTM) networks, which specialize in capturing relationships in sequential data (including DNA and RNA). Some tools combine several ML/DL approaches and may integrate other approaches (hybrid or *k-mer*-based).

**Table 1:** Summary of computational approaches to detect viral signals in sequence data

Approach	Basis	Methods	Advantages	Limitations
Sequence similarity (SeqSim)	Infers homology from local sequence alignment	BLAST-based searches, HMM profiles from phage gene databases (e.g. pVOGs)	High accuracy when close reference sequences exist	Poor detection of novel or divergent phages; dependent on reference database completeness
Hybrid	Combines similarity-based and homology-independent genomic features	Sequence similarity and agnostic features (like GC/AT skew, gene density, transcription direction, and tRNA presence)	Improved accuracy and flexibility; detects fragmented or novel phages	Computationally more complex; requires integration of diverse signals
*k-mer-*based	Identifies composition patterns using short *k-mer* frequencies	*k-mer* frequency profiling, composition clustering	Alignment-free; efficient detection of rearranged or unknown sequences	Sensitive to *k-mer* size and sequence quality; still somewhat reference-biased
Machine learning and deep learning (ML/DL)	Learns patterns from data using statistical models	RFs, SVMs, CNNs, and LSTMs; often use *k-mer* or protein features	Can detect novel viruses by learning complex, non-obvious patterns	Requires large, high-quality training data; model tuning and validation are non-trivial

## The evolution of phage prediction tools

Phage prediction tools emerged around the turn of the millennium to identify prophages in single bacterial genomes, at a time when available sequencing data were still scarce [19]. These early tools exploited simple composition-based signals [20], such as sudden shifts in GC content or dinucleotide relative abundance, to identify candidate prophage regions in host genomes [21–23]. However, their narrow scope made them unreliable for identifying low-abundance and cryptic prophages [19]. In the mid-2000s, a second wave of tools strongly enhanced prediction accuracy by integrating sequence composition analysis with homology-based methods. Phage_Finder [24], Prophage Finder [25], and Prophinder [26] integrated protein homology searches, tRNA detection, phage integration site prediction, and HMMs trained on viral genes. Despite these improved capabilities, their use was hampered by the limited diversity of available viral sequences and the technical expertise required for their implementation [19]. In response, tools such as the PHAST suite [19, 27, 28] implemented user-friendly web servers, which made *in silico* prophage prediction from closed single genomes accessible to many microbiologists and contributed to the suite’s high popularity. Moreover, PhiSpy [29] introduced hybrid approaches, the combination of sequence-similarity searches with sequence-agnostic features to improve the detection of novel and atypical prophages.

Yet, the advent of metagenomic sequencing completely changed the landscape of virus detection software, opening up unprecedented opportunities for phage discovery while exposing limitations in earlier tools designed for prophage detection in single, complete genomes. Metagenomic datasets are significantly larger and more taxonomically diverse, which requires more scalable virus detection methods that could also handle lowly covered and highly fragmented viral sequences. Early tools such as VirSorter [30] and MetaPhinder [31] extended detection to mixed-community data. VirSorter offered broad coverage with modular outputs but suffered from high false-positive rates [47]; and while MetaPhinder offered higher precision, it was constrained by its reliance on close similarity to known reference genomes, limiting its power to identify novel or mosaic phages [32].

The limitations of those initial tools, either prophage detectors constrained by known genome characteristics or early metagenomic tools restricted by reference similarity, boosted the development of a next generation of virus detection approaches starting from the latter half of the 2010s. These tools introduced conceptually distinct innovations to tackle key challenges:

- Kraken [33] and Kraken2 [34] forewent alignment altogether and used hash-based *k-mer* mapping, which improved scalability for fractured metagenomic data.
- VirFinder [35] (conventional ML) and DeepVirFinder [36] (CNNs) also use *k-mer* mapping but replace hand-tuned rules with ML and DL, respectively, further boosting sensitivity for novel or uncharacterized phages.
- MARVEL [37] tackled low-abundance viruses by making predictions from metagenome-assembled genomes (MAGs) using RF classifiers.
- VirMiner [38] combined ML-based classification with host prediction and gene annotation for deeper ecological insight.
- PPR-Meta [39], VIBRANT [32], Seeker [40], and Virtifier [41] employed different DL architectures (CNNs or LSTMs) to learn viral signatures from raw or protein-level data, especially suitable for mosaic or rearranged genomes.
- VirSorter2 [42] distinguishes itself through an ensemble ML approach that combines multiple phage-specific classifiers trained on viral and host genomic features, enabling robust and accurate predictions across a wide range of input types.
- PhaMer [50], finally, introduces a paradigm shift in phage detection by applying Transformer-based large language models (LLMs) to protein-tokenized phage contigs, enabling the capture of long-range dependencies and hidden sequence patterns characteristic of compositionally atypical or cryptic phages.

PhaMer was integrated into PhaBOX2 [43], a comprehensive and user-friendly pipeline that bridges multiple tools in an end-to-end workflow. The use of such integrated pipelines is a recent trend in the field aimed at fostering reproducibility, scalability, and accessibility. In summary, cutting-edge phage prediction software is increasingly defined by its ability to detect divergent, low-abundance, or structurally complex phages, even from noisy, fragmented metagenomes. This is a significant shift away from earlier static, sequence-similarity-based tools. Today, modern approaches incorporate dynamic, data-driven ML/DL algorithms that have been developed to handle the scale and complexity of large metagenomic datasets.

## A detailed step-by-step instruction guide

**Figure 3:**
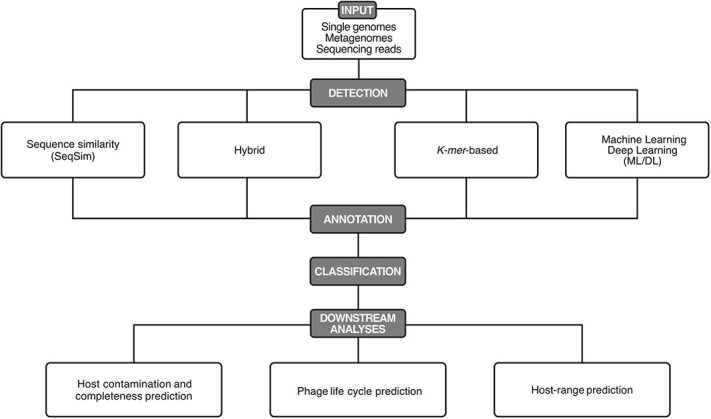
Workflow for phage detection and analysis. This outline reflects the key steps of phage detection, annotation, classification, and further downstream analyses.

Our goal is to offer guidance on navigating the expanding landscape of phage analysis tools used for single genomes and metagenomes. To this end, we cover four essential steps (Fig. 3): phage detection, annotation, taxonomic classification, and further downstream analyses (including quality of predicted phages, phage life cycle, and host prediction). We conclude by presenting integrated pipelines that streamline phage analysis by automating most or all of these steps.

### Step 1: Phage detection

#### General considerations

**Figure 4:**
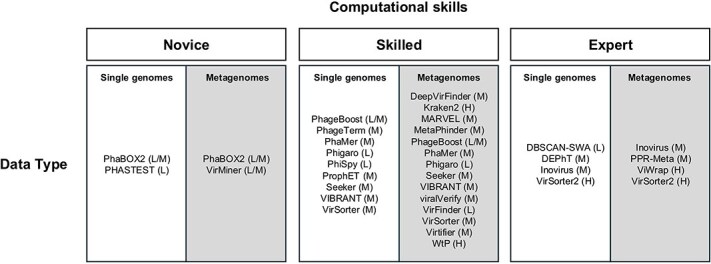
Classification of virus prediction tools by data type and user expertise. Tools are grouped based on their intended input (single genomes *versus* metagenomes) and anticipated user expertise. Approximate computational requirements are indicated in parentheses (L = low, M = medium, H = high; see also Table 3). Tools applicable to both single genomes and metagenomes are listed in both categories.

Most automated phage detection tools covered in this review operate on assembled contigs or genomes, e.g. in FASTA or annotated GBK formats. When working with assembled contigs, small genomic fragments can strongly hamper downstream analyses, including host prediction and viral core gene identification [44]. Thus, it is highly recommended to remove contigs <500 bp [35]. Several tools, including Kraken2 [34], VirMiner [38], and the pipelines PhaBOX2 [43] and ViWrap [45], all accept raw reads (FASTQ files). In particular, VirMiner [38] offers built-in modules for read pre-processing and classification, both of which are recommended. In the following, we present tools for phage discovery from metagenomes, then introduce prophage scanners for single genomes, and conclude with a brief section on filamentous phage detection. All prediction tools have been visually categorized (Fig. 4) and tabulated for data type, user expertise, and computational resource demands (Table 2, categories explained in Table 3).

**Table 2:** Summary table of phage prediction tools.

Tool	Expertise	Data type	Resources	Approach	Use case
DBSCAN-SWA [57]	Expert: CLI [115]	Single genomes	Low	Hybrid	Rapid batch processing and prophage detection
DeepVirFinder [36]	Skilled: CLI [120]	Metagenomes	Medium	*k-mer*, DL	CNN-based tool for viral sequence detection from metagenomes
DEPhT [58]	Expert: CLI [116]	Single genomes	Medium	Hybrid	Rapid batch processing and prophage detection with boundary detection (focus on *Mycobacterium*)
Inovirus [18, 59]	Expert: CLI [60]	Single- and metagenomes	Medium	Hybrid, ML	ML predictor for filamentous phages from assembled genomes
Kraken2 [34]	Skilled: CLI [121]	Metagenomes	High	*k-mer*	Hash-based taxonomic *k-mer* sequence classification with high resource (RAM) demands
MARVEL [37]	Skilled: CLI [122]	Metagenomes	Medium	Hybrid, ML	RF-based recovery of tailed phage candidates from metagenomic bins; focus on *Caudovirales*
MetaPhinder [31]	Skilled: CLI [123]	Metagenomes	Medium	SeqSim	Phage identification from metagenomes via BLAST searches against custom phage DB; also detects filamentous phages
PhaBOX2 [43]	Novice: Web [132] Expert: CLI [133]	Single- and metagenomes	Low, Medium	Hybrid, DL	Integrated workflow for phage identification with lifestyle, host, and taxonomy prediction from contigs with visual outputs
PhageBoost [56]	Novice: Web [134] Skilled: CLI [135]	Single- and metagenomes	Low, Medium	ML	RF-based prophage detection with read quality control, assembly, and functional annotation
PhageTerm [95]	Skilled: CLI [117]	Single genomes	Medium	Hybrid	Accurate phage termini and packaging inference (requires reads)
PhaMer [50]	Skilled: CLI [136]	Single- and metagenomes	Medium	DL	Deep-language-model-based tool for phage detection from metagenomes
PHASTEST [52]	Novice: Web [118]	Single genomes	Low	Hybrid	Rapid web-based prophage detection and annotation
Phigaro [54]	Skilled: CLI [137]	Single- and metagenomes	Low	Hybrid	Scalable, high-throughput prophage prediction and annotation
PhiSpy [29]	Skilled: CLI [53]	Single genomes	Low	Hybrid, ML	RF-based prophage detection from annotated genomes, with boundary refinement
PPR-Meta [39]	Expert: CLI [124]	Metagenomes	Medium	DL	CNN-based phage and plasmid prediction
ProphET [55]	Skilled: CLI [119]	Single genomes	Medium	SeqSim	Prophage prediction using an auto-updating reference database, is best for known phages
Seeker [40]	Skilled: CLI [138]	Single- and metagenomes	Medium	DL	Alignment-free phage detection based on LSTM-models
VIBRANT [32]	Skilled: CLI [139]	Single- and metagenomes	Medium	Hybrid, DL	Automated DL tool trained on protein signatures for virus detection, annotation, and life cycle prediction
viralVerify [49]	Skilled: CLI [125]	Metagenomes	Medium	ML	Filters viral contigs from metagenomic assemblies; low precision on single-genome prophage scans
VirFinder [35]	Skilled: CLI [126]	Metagenomes	Low	*k-mer*, ML	Fast alignment-free approach to detect viral sequences in metagenomes; biased to known phages
VirMiner [38]	Novice: Web [127], Skilled: CLI [128]	Metagenomes	Low, Medium	ML	Highly sensitive RF model for virus and host predictions with functional annotation
VirSorter [30]	Skilled: CLI [140]	Single- and metagenomes	Medium	Hybrid	*De novo* hybrid virus detection from metagenomes with custom probabilistic models
VirSorter2 [42]	Expert: CLI [141]	Single- and metagenomes	High	Hybrid, ML, DL	Highly modular ML/DL hybrid pipeline to detect DNA and RNA viruses in complex viromes
Virtifier [41] (Seq2Vec)	Skilled: CLI [129]	Metagenomes	Medium	DL	Viral contig identification from metagenomes based on LSTM classifiers; also, for contigs <500bp
ViWrap [45]	Expert: CLI [130]	Metagenomes	High	Hybrid, ML	Modular integrated workflow for phage identification, binning, classification, and host prediction
What the Phage (WtP) [112]	Skilled: CLI [131]	Metagenomes	High	Hybrid, ML, DL	Scalable phage identification and analysis pipeline; includes ML/DL

**Table 3:** Explanation guide of expected user expertise and computational resource requirements for different viral detection tools (as referred to in Table 2, Fig. 4).

Level	Definition	Explanations and examples
Required user expertise		
Novice	Minimal to basic bioinformatics exposure; intuitive web interface or GUI	Web/GUI: point-and-click usage, sequence selection, or upload
Skilled	Proficient with CLI (command-line interface)	CLI basics in Bash, GitHub, Conda, Python, or R
Expert	Experienced with automation, tool chaining, and high-performance computing (HPC)	HPC usage, Snakemake, Docker, workflow debugging
Required computational resources		
Low	Web, Standard computer (≤8 GB RAM, 1 CPU)	Ideal for casual, exploratory, or classroom use
Medium	Moderate workstation (8–32 GB RAM, multi-core CPU, moderate storage space)	Suitable for most genome and medium-sized metagenomic datasets
High	Requires server or HPC resources (>32 GB RAM; multiple threads for parallelization; large storage space)	For demanding high-throughput projects and complex workflows

#### Tool guide for analyzing metagenomes

We begin our tool guide with metagenomic phage detection tools, as this branch has become the fastest-growing field for viral bioinformatics. Tool performance can vary widely with input quality, contig fragmentation, and the viral/bacterial reference databases used [11]. To make informed decisions, users must rely on context-aware benchmarks [46–48]. Thorough benchmarks report the following standard metrics for tool cross-comparison:

- precision (fraction of predicted viral contigs that are truly viral),
- recall (fraction of all true viral contigs that are correctly recovered), and the
- F1 score (the balanced, harmonic mean of precision and recall).

**Figure 5:**
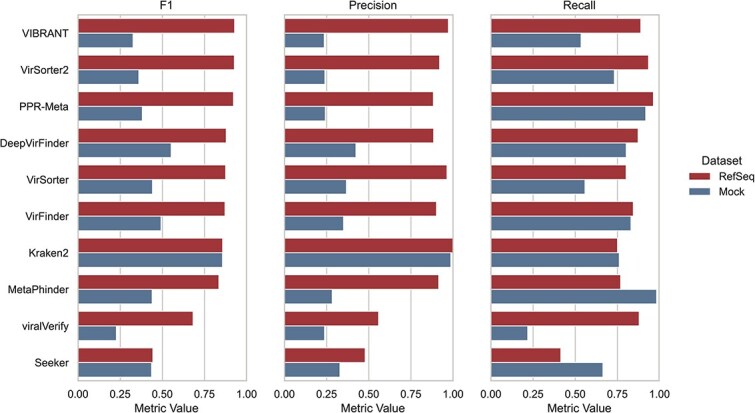
Benchmark performance of virus prediction tools on metagenomic datasets. Bar plots show the F1 score, precision, and recall of 10 metagenomic viral prediction tools, evaluated on either RefSeq-derived sequences (red, upper bar) or synthetic mock community data (blue, lower bar). Each bar represents the average performance for the respective tool and dataset. Tools are sorted by decreasing F1 scores on the RefSeq dataset for clarity. Performance metrics adapted from [47] under the terms of the Creative Commons Attribution 4.0 International License (http://creativecommons.org/licenses/by/4.0/).

Figure 5 summarizes results from the comprehensive benchmark *"Gauge your phage"* [47], which evaluated 10 widely used metagenomic virus detection tools on artificial contigs created from RefSeq genomes, previously sequenced mock communities, and randomly shuffled sequences. For RefSeq-derived sequences, the top performers were VIBRANT [32], VirSorter2 [42], and PPR-Meta [39], in that ranked order, with F1 scores higher than 90% (Fig. 5). These skilled-to-expert-level tools have high precision and recall at optimal conditions (for non-fragmented contigs up to 15 kbp length). VirSorter2 is especially well-suited to dealing with intricate viromes; however, its high flexibility trades off against longer runtimes compared to the other metagenomics tools. The less complex VIBRANT had much shorter run times and shows higher precision than VirSorter2 but had lower recall success. PPR-Meta is even faster than VIBRANT or VirSorter2 based on its resource-optimized DL classifiers; however, it also produced more false positives than both of the aforementioned tools.

In contrast, the more complex mock community dataset generally favored *k-mer*-based tools (Fig. 5). Here, the memory-intensive Kraken2 [34] had, by far, the highest F1-score across tools, which was carried by both excellent precision and recall. DeepVirFinder [36] ranked second due to a lower precision, but offers a highly resource-efficient alternative to Kraken2. As a consequence, *k-mer*-based methods are especially powerful for detecting phages in highly fragmented, contaminated, or low-abundance metagenomic data, such as environmental samples or ancient DNA. Their alignment-free nature allows for rapid detection of sequence composition patterns, even in the absence of close homologs, making them particularly effective when reference databases are incomplete or when sequence similarity is unreliable [33–36, 47].

Outside the top tier, VirSorter [30] delivered only moderate scores, especially on the more challenging mock-community data, and was clearly outperformed by its successor, VirSorter2. Likewise, VirFinder [35] was “easily handled” by its successor DeepVirFinder, whose convolutional-network model benefits from a much larger and more diverse viral training set. At the lower end of the spectrum, viralVerify (a module of MetaWRAP [49]) and Seeker [40] generally struggled with most benchmarks. Seeker also failed to represent both alpha- and beta-diversity in mock virome data, meaning it underestimated within-sample viral richness and between-sample community differences [47]. This makes Seeker unsuitable as a primary tool for viral ecology studies focused on diversity patterns or compositional structure. Among widely used tools outside of the scope of the benchmark, we want to single out two: MARVEL [37] and PhaMer [50]. MARVEL is a high-throughput tool intended for detecting free, tailed *Caudovirales* phages from metagenomic bins and is especially suitable for low-abundance and fragmented sequences when binning is feasible. In its original validation [37], MARVEL outperformed VirSorter and VirFinder in recall while maintaining similarly high precision, particularly on simulated MAGs. It is less suitable for detecting viruses from highly fragmented, unbinned contigs. PhaMer [50] utilizes a LLM for classifying phage contigs and is particularly effective at detecting cryptic and compositionally atypical phages. It achieved an F1-score of 0.93 on RefSeq-derived contigs and outperformed VirSorter, (Deep)VirFinder, Seeker, and PPR-Meta on mock metagenomic datasets [50]. While PhaMer requires high computational resources, this limitation is mitigated by its integration into the online workflow PhaBOX2 [43]. As a final note, averaging results from multiple prediction tools does not always improve accuracy, as many tools share overlapping reference biases and interdependent training data [46]. Therefore, tool outputs should be interpreted independently. Moreover, other factors, such as tool interface and computational resource demands, can be equally decisive (Tables 2 and 3, and Fig. 4) and should guide tool choice based on the dataset’s complexity and the user’s expertise.

#### Tool guide for prophage detection in single genomes

**Figure 6:**
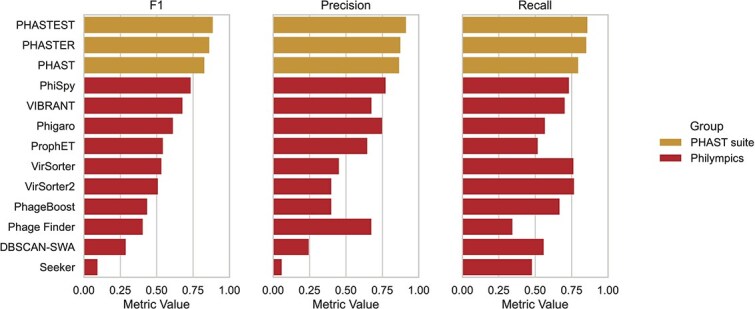
Benchmark performance of tools used for prophage detection in single genomes. Horizontal bar plots compare F1 score, precision, and recall (panels from left to right) across 13 tools. Orange, data adapted from the PHAST suite benchmark [52], licensed under a Creative Commons Attribution-NonCommercial License (https://creativecommons.org/licenses/by-nc/4.0/); red, data adapted from the *Philympics 2021* benchmark [51], licensed under a Creative Commons Attribution 4.0 International License (http://creativecommons.org/licenses/by/4.0/).

Compared to the metagenome-oriented tools described in the previous section, dedicated single-genome scanners offer higher efficiency and accuracy for identifying prophages in individual bacterial genomes. Figure 6 summarizes benchmark results from the *Philympics 2021* study [51] and is supplemented with performance data from the PHAST suite [52]. Among all evaluated tools, PHASTEST achieved the highest overall performance across all tested metrics, though it was run on a different dataset (Casjens-54) [20]. It is highly recommended for users who prefer GUIs and provides quick but sensitive open-reading frame (ORF) annotation. Analyses are typically complete within minutes per genome, with interactive visualizations of prophage locations in the output [52]. Within the Philympics benchmark, the updated version of PhiSpy [53] led the field. It offers robust precision and recall without relying on static reference databases. PhiSpy features RF classifiers on annotated genomes, includes refined prophage boundary detection, and is especially well-suited for skilled users who value flexibility and parameter control. Among other high-performing tools, Phigaro [54] offers robust throughput by combining Prodigal gene prediction with HMM-based pVOG annotation. ProphET [55] also performed well and is notable for including a self-updating reference database. At the lower end of the performance spectrum, PhageBoost [56] and the batch-processing tool DBSCAN-SWA [57] showed significant drops in precision and boundary resolution. Of note, metagenome-focused virus predictors performed poorly on these single-genome benchmarks, showing lower precision, longer runtimes, and poor phage boundary resolution. This highlights the importance of using tools designed for single-genome prophage detection. While not part of the performance benchmark, DEPhT [58] deserves mention for its precise prophage boundary detection. As always, tool choice should be guided by the specific research question and available computational resources (Tables 2 and 3, and Fig. 4).

#### Tool guide for detecting filamentous phages (*Inoviridae*)

Filamentous phages (*Inoviridae*) are characterized by rod-shaped or long proteinaceous filaments with a circular ssDNA genome of ~5–15 kb that can establish chronic infections. Because of their unique and diverse gene content, most computational approaches are inefficient at detecting their sequences from whole-genome shotgun sequencing data [18, 59]. The ML tool Inovirus [60] implements a two-step pipeline specifically designed for this purpose. In the first step, the program Inovirus_detector scans for conserved *Inoviridae* marker proteins (especially pI-like proteins) using HMMs. In the second step, an RF classifier detects other characteristic *Inoviridae* features, such as small structural proteins. These predictions are then passed to the Inovirus_classifier module, which refines the taxonomic ranking of candidate sequences within *Inoviridae*, based on conserved protein clusters. This approach enables the automated discovery and taxonomic classification of inoviruses. The authors reported high recall and precision values of 92.5% and 99.8%, respectively, on a manually curated reference set [18, 60]. Of note, Virsorter2 [42] is also capable of identifying *Inoviridae*, as it includes pI-like proteins in its viral marker set.

Once high-confidence phage regions have been identified, users typically proceed to annotate and characterize the predicted viral genes. We describe this step in the following section.

### Step 2: Phage gene prediction and annotation

#### Phage gene prediction

While tools such as GLIMMER [61], GeneMarkS [62], and Prodigal [63] were originally designed for application to bacterial genome annotation, they are frequently applied to phage genomes as well. However, their performance is limited by the compact and atypical architecture of phage genomes, which typically feature more overlapping, short, and embedded genes [64–66]. To address this, the graph-based PHANOTATE [67] was developed specifically for the compact nature of phage genomes. A benchmark with 2133 complete phage genomes showed that PHANOTATE predicted more genes than GLIMMER, GeneMarkS, and Prodigal and had an ~82% agreement with genes predicted by at least one of these tools [67]. Importantly, ~6% of its predictions were unique but mostly evolutionarily conserved. This suggests that PHANOTATE can uncover functional proteins that are not detected with standard approaches. As a best-practice recommendation, the outputs from various gene prediction tools should be compared. To this end, the comparative platforms Phage Commander [68] and MultiPhATE2 [69] assess consensus calls, visualize overlaps, and help select the most plausible gene models.

#### Functional gene annotation

Unlike in cellular organisms, prokaryotic viruses lack a universal common ancestor, and their proteins exhibit limited conservation levels. Therefore, only a minority of phage genes have known functions, which hampers the functional annotation of newly detected phage genes. To address this, Pharokka [70] integrates the prokaryotic virus remote homologous groups (PHROG) database [71], which clusters viral proteins into orthologous groups based on remote homology and manual curation. Paired with the PHANOTATE gene caller, Pharokka provides appropriate prediction and meaningful annotation for newly identified phages. For users who prefer web-based tools, PhANNs [72] offers an artificial neural network (ANN) ensemble to rapidly classify proteins into 10 structural classes. Finally, highly fragmented metagenomic assemblies present a significant challenge for standard gene callers. In this context, Balrog [73], which employs temporal CNNs, demonstrates strong performance by significantly reducing the number of hypothetical gene predictions. It effectively retains well-conserved genes while removing spurious ORFs, which improves confidence in both gene prediction and downstream annotation.

### Step 3: Taxonomic classification

Historically, viruses were primarily classified based on phenotypes, e.g. traits such as tail morphology or capsid shape. Because such morphocentric groupings often lacked monophyly, the international committee on taxonomy of viruses (ICTV) [74] redefined viral taxonomy to be based on genomic and proteomic information. At higher taxonomic ranks (family, order, and class), classification is now done based on viral hallmark genes and whole-proteome comparisons. This approach is implemented by several programs, comprising VICTOR [75] and ViPTree [76], which both conduct whole-proteome phylogenetic inference; vConTACT2 [77], which groups viruses in terms of common protein clusters; GRAViTy [78], which integrates genomic architecture and protein profile HMMs; and VirClust [79], which uses adaptive homology models for proteins to identify taxonomic clusters across taxonomic levels without sacrificing sensitivity and specificity.

At lower taxonomic levels (genus and species), whole-genome or individual-gene alignments remain essential. However, phages frequently lack a common core genome due to high recombination frequencies or genomic mosaicism [80]. This complicates traditional phylogenetic classification. Clustering based on intergenomic nucleotide identity can circumvent this limitation. For example, VIRIDIC [81] clusters phages based on user-defined similarity levels, while ClassiPhage and ClassiPhages 2.0 [82, 83] utilize HMMs and ANNs to classify phages by conserved features.

However, taxonomic classification of novel or highly divergent phages remains challenging. Most lack sufficient similarity to reference sequences, and their modular genome structures impede the application of conventional classification methods. To better reflect evolutionary relatedness in these cases, several studies have adopted genome-wide similarity metrics as complementary approaches: average nucleotide identity (ANI) [84] and weighted gene repertoire relatedness (wGRR) [80].

ANI calculates the average nucleotide identity of orthologous regions of genes between two genomes. It is achieved by fragmenting genomes, matching homologous regions, and averaging nucleotide similarity. ANI accurately distinguishes phages at the genus or species levels but is less effective for highly recombinant or mosaic genomes, where alignable regions can be sparse.

In contrast to ANI, which relies on nucleotide-level similarity, wGRR establishes similarity at the protein level through the detection of reciprocal best hits between genomes and their weighting based on both sequence identity and alignment coverage. This protein-centric approach enables the estimation of evolutionary relatedness to be robust even when nucleotide homology is fragmented or low. While not a taxonomic method *per se*, wGRR is best applied for clustering phages based on shared gene content and evolutionary patterns, particularly if core genes are lacking or disrupted by recombination.

### Step 4: Further downstream analyses

Following detection and taxonomic classification, different types of downstream analysis can provide key functional, ecological, and evolutionary information. These encompass sequence quality analysis, life cycle prediction, and the prediction of potential hosts, especially essential for phage-therapeutic design and viral ecology studies. We recommend three main categories: (i) quality assessment, and prediction of (ii) phage life cycle, and (iii) phage host. Other downstream analyses beyond this guide are core gene prediction and gene transfer [80, 85], viral density estimation (VIRMOTIF [86]), or functional potential prediction of viral communities [87]. Instead of a comprehensive list, we prefer to provide the beginner with a helpful overview of frequently used tools and best practices.

#### Quality assessment of phage genomes

Assuring high quality of novel genome assemblies is crucial for reliable annotation, taxonomic classification, and ecological interpretation. This is because incomplete or contaminated genomes can obscure significant viral functions or lead to incorrect taxonomic classification. To circumvent these issues, CheckV [44] is the most suitable software for precise assessment of host contamination and genome completeness. It uses reference-based scoring for known phages, HMM-based inference for novel viruses, GC content, and terminal repeat detection to determine completeness level and contamination status. It reports completeness values as a percentage of complete viral genome for each contig. In addition, other tools also measure viral completeness with different approaches: VIBRANT [32] scans for characteristic viral proteins; *viralComplete* [88] employs reference-length and content; PHASTEST [52] offers ORF-level completeness scores; and Phables [89] reconstructs fragmented metagenomic assemblies into genomes using flow-based graph modeling, a unique feature among existing tools. Completeness estimates are reference-coverage dependent and can miss novel genomes. Therefore, we recommend visually inspecting all datasets.

#### Life cycle prediction

Phage lifestyle prediction is a reflection of their ecological roles and therapeutic potential. However, most of the current methods predict lysogeny based on conserved markers or a positive hit to known integrases, a characteristic that novel viruses might not have. Furthermore, if only genome structure is considered, it is impossible to determine whether a prophage is biologically active. To ensure strong inferences of phage lifestyles, genomic predictions should be complemented by contextual data, such as gene expression or culture-based strategies. For automated prediction, the tool landscape offers a variety of different approaches, e.g. PHACTS [90] or BACPHLIP [91]. PHACTS employs RF classification to cross-match phage genomes with a reference database of phages whose known life cycles have been characterized, and BACPHLIP [91] distinguishes between temperate and virulent phages according to their conserved protein domains. Lytic or temperate life cycles can further be predicted for highly fragmented phages derived from short-contig assemblies (PhaTYP [92]) or metaviromes (DeePhage [93] or PhagePred [94]). Also, PhageTerm [95] can be employed to predict the packaging mechanisms when both sequencing reads and an assembly are available.

#### Host prediction

The accurate inference of a phage’s host range is crucial for any meaningful ecological interpretation, but also for technical considerations such as microbiome engineering and assessing therapeutic potential for phage therapy. Host ranges are traditionally assessed in the laboratory, which is both time-consuming and restricted to culturable bacteria. *In silico* host prediction is therefore now critical, especially for large-scale metagenomic data for which cultured hosts do not exist for viral sequences. *In silico* host prediction methods fall broadly into two categories.

#### Host prediction: Database-driven matching

These methods can be further classified into repositories of documented or predicted phage-host interactions (PHI-base [96], ViralHostRangeDB [97], and MVP [98]) and predictive computational approaches that infer a host from an input phage sequence. For the latter host prediction tools, they must strike a good balance between recall and false discovery rate (FDR). Here, PHISDetector [99] and VirHostMatcher-Net [100] show favorable recall values for the task, but they also reported unfavorably high FDRs of >10%. On the other hand, the supervised tool iPHoP [101] gives low FDRs coupled with high recall values for known and even novel phages at the genus level. Technically, it employs an automated approach that integrates database comparison with genome pattern analysis to simplify host prediction. Phage hosts can also be inferred by aligning the query phage with a database of known phage-host pairs, e.g. RaFAH [102], or by analysis of sequence alignment patterns, which can reveal prophage or CRISPR integration (using SpacePHARER [103]).

#### Host prediction: Alignment-free sequence feature models

These approaches analyze oligonucleotide usage patterns or trained sequence features to infer host identity without alignments. These include a collection of different tools, which determine the host genome *k-mer* frequencies relative to the phage genome, e.g. WIsH [104], PHIST [105], DeepHost [106], HostPhinder [, 107], and PHP [108]. Among them, the Prokaryotic virus Host Predictor (PHP) [108] is the most accurate at the genus level. It excels in situations where alignment-based methods fail and it features flexible host prediction from fragmented viral genomes and is particularly effective in predicting hosts from challenging metaviromes. Two accurate alternatives to PHP available for metaviromes are HoPhage [109], which features both a Markov-chain model and a DL-method for host genus prediction, and CHERRY [110], which combines proteome- and genome-derived feature graphs. To achieve optimal results, researchers are advised to cross-validate predictions among complementary tools and include ecological metadata where available.

### Integrated pipelines

Phage discovery and downstream analysis is a step-by-step approach that includes the successive or parallel employment of different dedicated tools. In response, several groups have designed integrated virus analysis pipelines that bundle tools into workflows, comprising all or most of the steps outlined in this review (Table 3). Here we provide several examples that demonstrate the range of approaches currently available. PhageCompass (https://phagecompass.ku.dk) is a web application built by an international collaboration for translational phage therapy. It integrates several evaluation tools (including PhageBoost [56]) into a structured and easily accessible web interface, supporting open access and educational outreach. MetaPhage [111] is a Nextflow-based modular pipeline for expert users. It facilitates virus mining from metagenomic data through a multi-step process including read classification, assembly, and virus prediction through an ensemble of tools (including Phigaro, VIBRANT, VirFinder, and VirSorter). The pipeline “What the phage” (WtP) [112] is a reproducible and scalable NextFlow workflow for expert users comprising multiple phage detection tools (including VirFinder, PPR-Meta, VirSorter1/2, Seeker, MetaPhinder, DeepVirFinder, and VIBRANT) with subsequent virus annotation and classification (using Phigaro) and offers user-friendly summaries in chart and table format. Finally, PhaBOX2 [43] is suitable for both single genomes and metagenomes and offers a highly accessible, web server-based pipeline. It takes contigs/sequences in FASTA format and runs virus identification (PhaMer [50]), taxonomic classification (PhaGCN [113]), host and lifestyle prediction (CHERRY/HostG [110] and PhaTYP [92]), contamination and provirus integration screening, vOTU grouping, marker gene-based phylogenetic tree inference, and viral protein annotation using recent databases via ICTV 2024. Expert users can run PhaBOX2 locally using a command-line interface (CLI). In summary, workflows simplify the manual overhead of linking the outputs of multiple tools and produce formatted outputs that can aid reproducibility and interpretation, critical assets in large-scale virome studies and translational applications such as phage therapy.

## Conclusion

The advent of new computational ML and DL methods has significantly elevated the speed, accuracy, and sensitivity of virus prediction. Nevertheless, significant challenges persist, such as the identification and taxonomic placement of rare or uncommon phages or the discrimination of closely related viral genomes in highly complex metagenomic data. With the surging number of new bioinformatic phage tools and acknowledging that no single tool represents the optimal global approach for tackling all research questions, scientists increasingly must pair analytical approaches to their specific questions. This guide seeks to help that process by supporting researchers to make informed, capable decisions in aid of their goals and abilities. As the field of viral signal detection in large metagenomic datasets continues to evolve rapidly, our review is a mere snapshot of this ongoing development. We do hope, though, that our historical treatment of the various underlying algorithms will enable users to better grasp and categorize new tools as they emerge. It is essential to harness the full potential of the latest tools, and so we hope that our guide will support phage explorers in their quest to discover novel phage elements from (meta)genomic datasets. In the future, tool design will likely integrate ecological background, metadata standards, and gene-sharing network approaches. For instance, clustering algorithms based on gene sharing, such as vConTACT2 [77], effectively group new viruses irrespective of their taxonomy. Concurrently, initiatives such as MIUViG [114] are setting the necessary metadata standards to improve reproducibility in viral ecology research. Finally, advanced host prediction programs and ML/DL-models that have been trained on ecological or temporal patterns will likely bridge the gap between detection and interpretation.

**Key Points**

- The number and diversity of computational tools for predicting prokaryotic viruses from single genomes and metagenomic data have rapidly expanded over the past decade, reflecting both technical innovation and growing interest in viral applications like phage therapy.
- Without claiming to be exhaustive, a wide range of state-of-the-art phage prediction tools are discussed and critically evaluated.
- A step-by-step guide is proposed that covers and critically assesses tools for phage prediction, gene annotation, taxonomic classification, and more.
- Since user input data can vary and sequence databases differ, it’s essential to evaluate how well each tool works under different scenarios using reliable statistical measures and consistent benchmarks, which are discussed for both metagenomes and single genomes.
- In conclusion, *in silico* phage prediction provides valuable, testable hypotheses about phage biology and taxonomy, integration sites, and lifestyle traits, which all should be validated experimentally wherever possible.

## Data Availability

No new data were generated or analysed in support of this research.
